# BOLD-CSF dynamics assessed using real-time phase contrast CSF flow interleaved with cortical BOLD MRI

**DOI:** 10.1186/s12987-024-00607-8

**Published:** 2024-12-24

**Authors:** Emiel C. A. Roefs, Ingmar Eiling, Jeroen de Bresser, Matthias J.P. van Osch, Lydiane Hirschler

**Affiliations:** 1 C.J. Gorter MRI Center, Department of Radiology, Leiden University Medical Center, Leiden, The Netherlands; 2 Department of Radiology, Leiden University Medical Center, Leiden, The Netherlands

**Keywords:** CSF flow, Real-time phase contrast, Bold, Glymphatic system, Brain clearance, Bold-CSF coupling

## Abstract

**Background:**

Cerebrospinal fluid (CSF) motion and pulsatility has been proposed to play a crucial role in clearing brain waste. Although its driving forces remain debated, increasing evidence suggests that large amplitude vasomotion drives such CSF fluctuations. Recently, a fast blood-oxygen-level-dependent (BOLD) fMRI sequence was used to measure the coupling between CSF fluctuations and low-frequency hemodynamic oscillations in the human cortex. However, this technique is not quantitative, only captures unidirectional flow and is sensitive to B0-fluctuations. Real-time phase contrast (pcCSF) instead measures CSF flow dynamics in a fast, quantitative, bidirectional and B0-insensitive manner, but lacks information on hemodynamic brain oscillations. In this study we propose to combine the strengths of both sequences by interleaving real-time phase contrast with a cortical BOLD scan, thereby enabling the quantification of the interaction between CSF flow and cortical BOLD.

**Methods:**

Two experiments were performed. First, we compared the CSF flow measured using real-time phase contrast (pcCSF) with the inflow-sensitized BOLD (iCSF) measurements by interleaving both techniques at the repetition level and planning them at the same location. Next, we compared the BOLD-CSF coupling obtained using the novel pcCSF interleaved with cortical BOLD to the coupling obtained with the original iCSF. To time-lock the CSF fluctuations, participants were instructed to perform slow, abdominal paced breathing.

**Results:**

pcCSF captures bidirectional CSF dynamics with a more pronounced in- and outflow curve than the original iCSF method. With the pcCSF method, the BOLD-CSF coupling was stronger (mean cross-correlation peak increase = 0.22, *p* = .008) and with a 1.9 s shorter temporal lag (*p =* .016), as compared to using the original iCSF technique.

**Conclusions:**

In this study, we introduce a new method to study the coupling of CSF flow measured in the fourth ventricle to cortical BOLD fluctuations. In contrast to the original approach, the use of phase contrast MRI to measure CSF flow provides a quantitative in- and outflow curve, and improved BOLD-CSF coupling metrics.

**Supplementary Information:**

The online version contains supplementary material available at 10.1186/s12987-024-00607-8.

## Background

In the past decade, cerebrospinal fluid (CSF) has been proposed to play a central role in brain homeostasis beyond its traditional functions of cushioning and immune surveillance. CSF mobility along perivascular spaces is now thought to remove harmful substances and thus play an important role in brain clearance [1]. Although the exact mechanisms of CSF-mediated brain clearance remain debated, increasing evidence suggests that large amplitude vasodilation (either from neuronal activity, naturally occurring vasomotion or induced by respiration) drives perivascular clearance [2–4]. This process is likely due to CSF displacement occurring as a result of cerebral blood volume changes that push CSF in and out of the neurocranium (Monro-Kellie doctrine) [5].

Recently, a direct coupling between CSF fluctuations in the fourth ventricle and low-frequency hemodynamic oscillations (~0.05 Hz in sleep, ~0.25 Hz in wake) in the human cortex was found using a fast resting-state blood-oxygen-level-dependent (BOLD) fMRI sequence [2, 6]. With this approach, it was shown that strong, anti-correlated waves in BOLD signal and CSF flow are present during deep sleep, which can be interpreted as increased brain clearance activity [7]. The main concepts behind this MRI sequence are two-fold: first, the sequence provides a BOLD measurement in brain tissue that is dependent on vasodilation; second, by using a short repetition time, MRI signal from static tissue will be saturated, which sensitizes the signal at the bottom slice to new CSF inflow into the fourth ventricle (later referred to as iCSF). However, iCSF is not quantitative, cannot capture caudal CSF outflow, and is sensitive to B0 fluctuations.

On the other hand, real-time phase contrast CSF flow measurement (pcCSF) [8–12] provides a fast, quantitative, bidirectional, B0-insensitive estimation of CSF flow dynamics. Besides measuring CSF inflow towards the cerebral aqueduct, quantitative phase contrast imaging can also measure CSF outflow towards the foramen magnum and spinal canal. However, with real-time phase contrast no information is obtained on the vasodilatory processes inside the brain. Therefore, it only provides insights into CSF dynamics without informing us on its coupling to cerebrovascular processes as its driving force.

The new concept of the current study is to combine the strong points of the two approaches by interleaving each repetition of a cortical BOLD acquisition with a real-time phase contrast MRI measurement in the fourth ventricle, thereby allowing to quantify the interaction between CSF flow and cortical BOLD responses.

The aim of this study was two-fold: (1) Compare CSF flow measured using pcCSF to the iCSF signal by acquiring both techniques at the same location using repetition-time interleaving; (2) Study BOLD-CSF coupling obtained using the novel pcCSF interleaved with cortical BOLD MRI.

## Methods

### Participants

A total of 8 healthy participants (female = 5, mean age = 27 ± 4 years) were scanned at 7 Tesla (Philips Achieva, Best, The Netherlands) using a dual-transmit coil and a 32-channel receive head coil (Nova Medical, Wilmington, MA, USA). The experimental protocol was approved by the ethics committee of the Leiden University Medical Center under authorization number P07.096 and conducted according to the principles of the 2013 Declaration of Helsinki. All participants signed informed consent prior to participation.

### Scan interleaving

In this study, pcCSF and BOLD-iCSF scans were interleaved at the repetition level using a Philips patch [13]. The handover from one sequence to another was done after one real-time phase contrast measurement (including opposite bipolar flow encoding) and after all slices of the BOLD sequence were acquired. Preparation phases of both sequences are also acquired back-to-back preceding the actual imaging sequence. This method enabled combined measurements of both CSF (in)flow and hemodynamic BOLD oscillations at a sampling rate of less than 450 ms per image pair.

### In vivo experiments

**Fig. 1:**
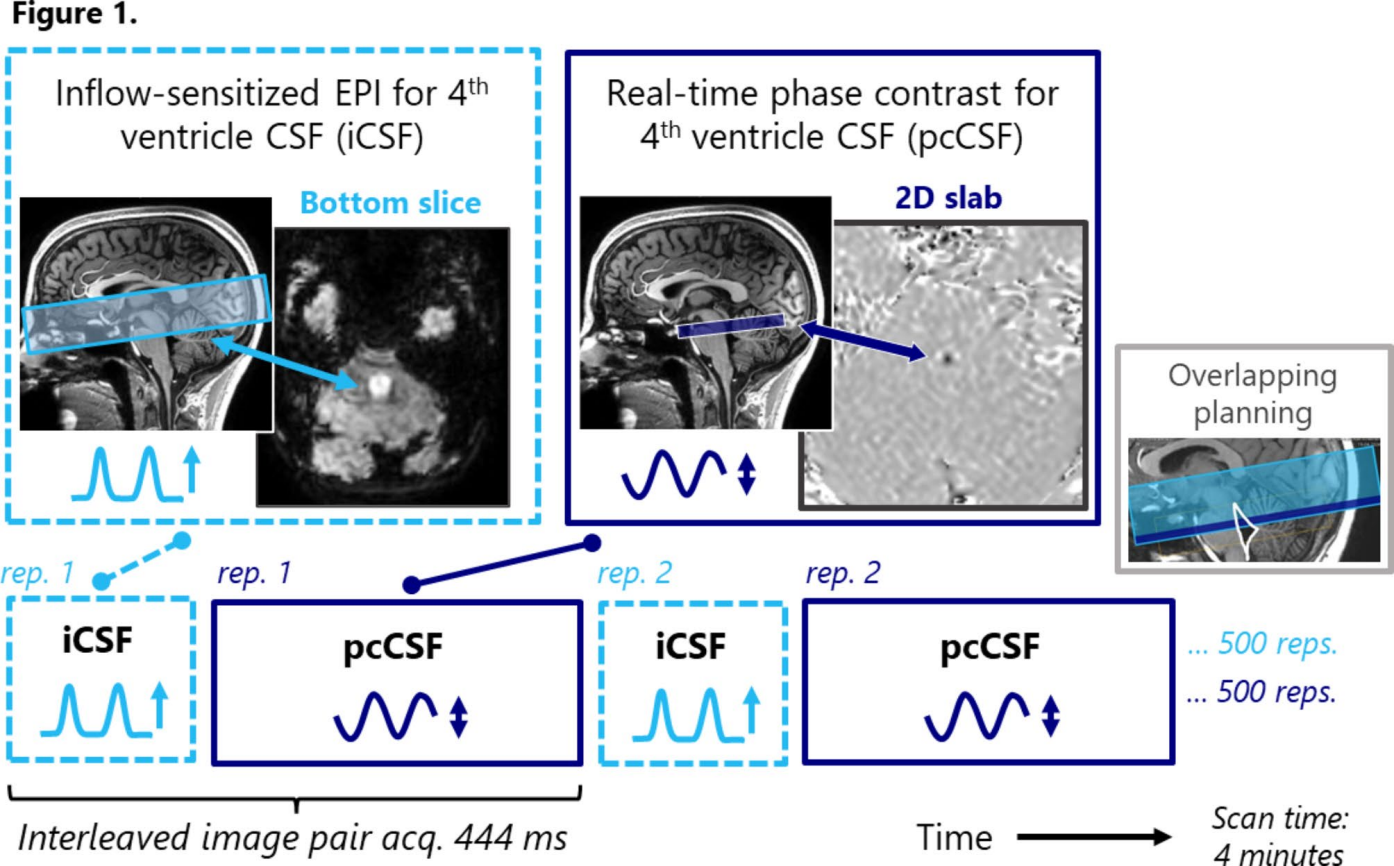
Scan set-up Experiment 1. Interleaving each repetition of pcCSF and iCSF at the same fourth-ventricle location results in a combined dynamic scan time under 450ms, i.e. every *n*-th repetition of iCSF scan is followed by the *n*-th pcCSF scan. A total of 500 repetitions were acquired per scan type. The purpose of this experiment is to compare the two techniques during the exact same physiological conditions. Acq.=acquisition, rep. = repetition number

**Fig. 2:**
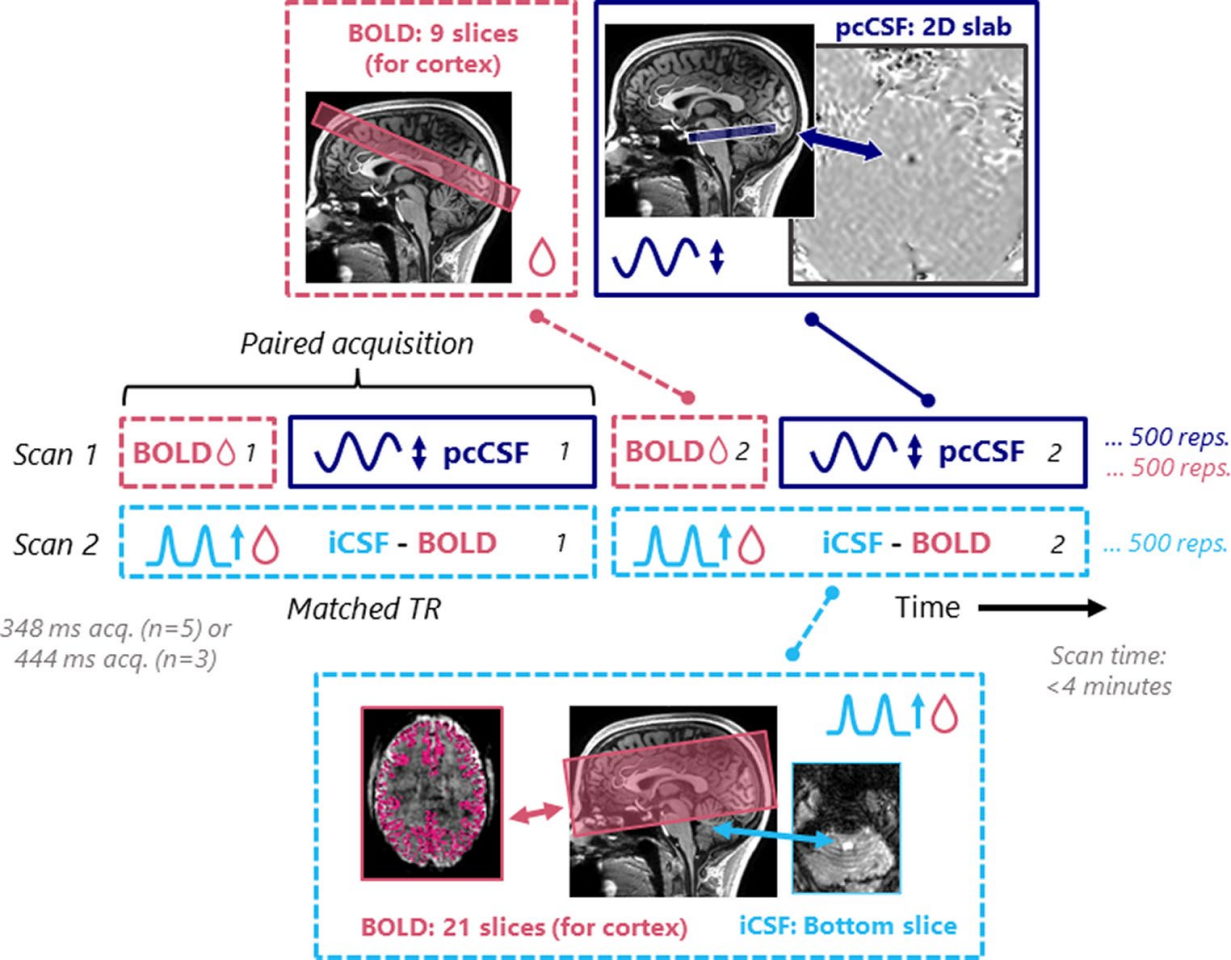
Scan set-up Experiment 2. Interleaving of pcCSF and cortical BOLD MRI for comparison to the standard iCSF for BOLD-CSF coupling. The repetition time (TR) of the iCSF is matched with the interleaved pair acquisition time of the BOLD-pcCSF. Acq. = acquisition, 2D = two-dimensional, rep. = number of repetitions

Two experiments were performed to compare the new approach of interleaving phase contrast with cortical BOLD MRI to the original approach of BOLD-iCSF imaging. In Experiment 1, a direct comparison was performed between the pcCSF and the iCSF signals by interleaving the measurements at the repetition level with the same fourth ventricle location. This experimental design is visualized in Fig. 1. Secondly, BOLD-CSF coupling was measured by interleaving the pcCSF and cortical BOLD scan. This method was compared to an iCSF scan, as visualized in Fig. 2. In this experiment iCSF and pcCSF measurements are not interleaved and thus performed under slightly different physiological conditions. Therefore, a breathing task was employed to impose comparable CSF flow patterns. The same breathing task was also employed in Experiment 1 to enhance CSF signal fluctuations in both scans and compare over breathing cycles between scan types.

#### Paced breathing task

Participants performed a paced breathing task to: (1) time-lock the CSF dynamics for all scans and participants to be able to compare across methods and participants; and (2) to enhance CSF flow fluctuations [9, 14]. The breathing task consisted of 30 s free breathing followed by 2.4 min of visually guided paced breathing at 0.1 Hz (5 s in – 5 s out, i.e. six cycles per minute). At the end of the 30 s rest period, a countdown was shown to inform the participant that the paced breathing task was about to start.

Visual guidance, showing an inflating and deflating light-blue filled circle within a black outline (showing maximal inhalation), was projected onto a screen viewed through a mirror mounted on the coil. Participants were asked to perform abdominal breathing and to restrict head motion as much as possible. In between breathing tasks, the participants got rest to adjust to their normal breathing rhythm.

Paced breathing at 0.1 Hz affects BOLD signal changes by altering cerebrovascular reactivity via changes in arterial CO2 [15]. The underlying changes in blood volume will lead to CSF flow in the fourth ventricle. Moreover, paced breathing causes negative thoracic pressure during inspiration. This will draw venous blood into the thorax resulting in increases in BOLD-signal and flow of CSF into the neurocranium as it fills the small temporary drop in intracranial pressure.

#### Experiment 1; pcCSF interleaved with iCSF

In Experiment 1 (*n* = 3), the interleaved pcCSF and iCSF scans were both planned at the fourth ventricle (Fig. 1), to directly compare CSF fluctuations measured with the unidirectional iCSF and the bidirectional pcCSF sequence. 500 interleaved repetitions were acquired with a combined repetition time of 444 ms.

#### Experiment 2; BOLD-CSF coupling comparison

In Experiment 2 (*n* = 8), real-time pcCSF was interleaved with a cortical BOLD scan of 9 slices to study BOLD-CSF coupling and compare it with the BOLD-iCSF method [6] (Fig. 2). As for three participants, the TR was longer (444 ms instead of 348 ms), the timeseries of iCSF and pcCSF were temporally resampled (interpolated) from 444ms to 348ms.

### Acquisition parameters

For the real-time phase contrast measurements, the scanning parameters were: a spoiled flow-compensated fast field echo sequence with FOV = 110 × 110 mm (APxRL), slice thickness of 3 mm (Experiment 1) or 10 mm (Experiment 2), resolution = 2 × 2 mm (reconstructed at 1 × 1 mm), SENSE (RL) = 3, partial Fourier = 0.7, TR/TE = 11.55/8.0 ms (Experiment 1) or TR/TE = 7.8/5.7 ms (Experiment 2), FA = 4°, and V_enc_ (foot-head) = 3 cm/s (Experiment 1) and 5 cm/s (Experiment 2), resulting in a measurement time of 300 ms for Experiment 1 and 202–204 ms for Experiment 2, depending on slab angulation.

When interleaving BOLD with the phase contrast scan, the imaging parameters were: FOV = 230 × 190 × 27 mm (APxRLxFH), resolution = 2.5 × 2.5 mm, slice thickness = 3 mm, SENSE = 3, simultaneous multi-slice (SMS) factor of 3, FA = 30°, EPI factor = 31, SPIR fat suppression, TR/TE = 144/22 ms. Pairs of 500 repetitions were acquired interleaved for both scans with a combined repetition time of 444 ms for Experiment 1 and 348 ms for Experiment 2.

For the standard iCSF method, the parameters were: FOV = 190 × 230 × 63 mm^3^, resolution = 2.5 × 2.5 mm, slice thickness = 3 mm, SENSE = 2, SMS = 3, FA = 30°, EPI factor = 31, SPIR fat suppression, TR/TE = 348/22 ms.

All scan sessions included a T1-weighted scan for planning and anatomical reference for post-processing, using a 3D-MPRAGE, resolution = 0.90 mm^3^ isotropic, TR/TE = 4.2/1.92 ms, FA = 7°, inversion time = 1300 ms. Respiration and cardiac cycles were continuously recorded by a respiratory belt and pulse plethysmography, respectively.

A volume shim was used for all phase contrasts scans, and a 3^rd^-order full brain shim was performed for the iCSF and BOLD scans. Reconstruction of the phase contrast was performed using the scanner software and consisted of a complex subtraction of both polarity acquisitions followed by concomitant field and 2D quadratic phase correction to correct for background phase changes. Separate scans with reversed phase encoding polarity were acquired for geometric distortion correction of the BOLD EPI scans (not acquired in 2 participants).

### Data analysis

#### Data pre-processing

Fourth ventricle masks were manually drawn on the standard deviation over time of the pcCSF scan and on the bottom slice of the non-motion corrected iCSF scan. The CSF masks were drawn conservatively to not include edge voxels that might shift in and out of the mask during motion. The iCSF timeseries were divided by the mean of their lower 30% of timepoints to convert these traces to percent signal change that represents CSF inflow.

The BOLD-iCSF scan was slice-time corrected to the bottom slice readout moment, using FSL *slicetimer*. For BOLD-pcCSF, a static offset of −174 ms or −222 ms (depending on the pcCSF TR) was applied instead, to align the middle of both interleaved acquisitions. Next, BOLD acquisitions were motion-corrected with three rotation, translation and zoom degrees of freedom using FSL *MCFLIRT* and corrected for EPI geometric deformations by combining them with a separate inverse phase encoding polarity scan and applying an FSL *TOPUP* warp [16].

Cortical masks were generated from the full brain T1-weighted images with FreeSurfer *– recon all* [17] by combining the right and left ribbons, resliced to the native T1-space using AFNI [18], and registered and resliced to the middle image of the motion-corrected BOLD timeseries using SPM12 [19]. The cortical grey matter probability map was masked using a threshold of 0.9 to create a cortical grey matter mask. The resliced mask was overlaid onto the preprocessed BOLD images to generate a mean global timeseries (termed gBOLD). The gBOLD timeseries were normalized by their total mean for conversion to percent signal change.

#### BOLD-CSF coupling analysis

BOLD-CSF coupling analysis was performed similarly to prior work [2, 6]. iCSF signals were spline-detrended. For the BOLD-iCSF analysis, the derivative of the gBOLD was computed and multiplied by −1 (later referred to as $\:-\frac{d}{dt}gBOLD$). Negative values were set to zero to account for the fact that the iCSF method only captures CSF inflow. For BOLD-pcCSF analysis, as the sequence captures both in- and outflow of CSF, $\:-\frac{d}{dt}gBOLD$ was computed without setting negative values to zero. Lastly, all signals were low-pass filtered at 0.15 Hz to better assess low-frequency fluctuations driven by the 0.1 Hz paced breathing paradigm and to filter out cardiac driven pulsations.

BOLD-CSF coupling, i.e. the signal coherence between $\:-\frac{d}{dt}gBOLD$ and iCSF or pcCSF, was computed using the *xcorr* function in MATLAB 2021a (MathWorks, Natick, MA, USA) with lags ranging from −8.7 s to +8.7 s with steps of 0.348 s. The maximum cross-correlation magnitude, *R*, was defined as the positive peak closest to lag *t =* 0 s. It reflects the highest signal coherence between the pcCSF/iCSF and $\:-\frac{d}{dt}gBOLD$ fluctuations. The lag of the peak *R* was extracted as well.

### Statistical analysis

To test whether the pcCSF method produces better BOLD-CSF coupling than iCSF, both the magnitude and lag of the cross-correlation peaks were compared between the iCSF and pcCSF scan pairs of each participant using Wilcoxon signed rank tests. Lastly, differences in cumulative net flow in the fourth ventricle between the three conditions (30 s free breathing prior to paced breathing, during paced breathing, and resting state), were tested using the Wilcoxon signed rank test. A significance threshold of *p* <.05 was chosen. All reported values are mean ± standard deviation unless stated otherwise.

## Results

### Experiment 1; pcCSF interleaved with iCSF

**Fig. 3:**
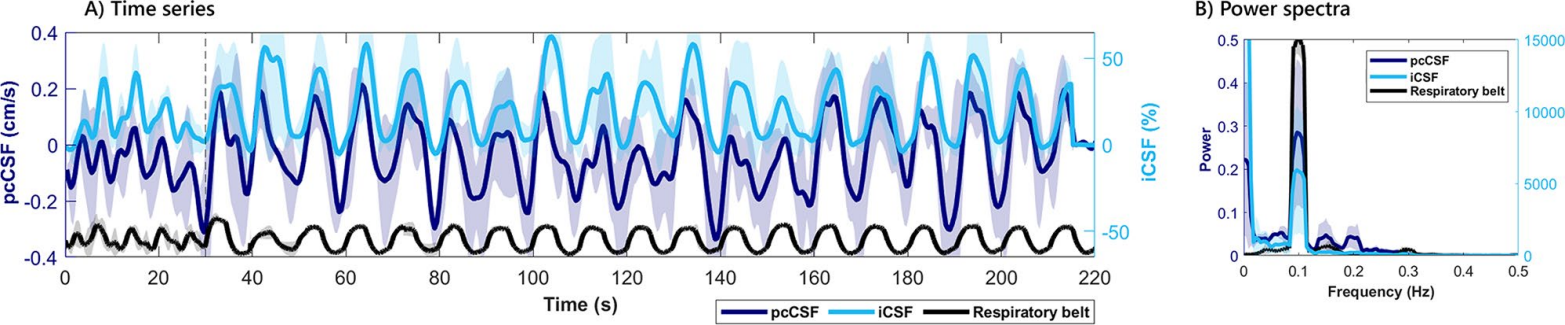
Interleaved pcCSF and iCSF scans at the fourth ventricle during paced breathing. Both the pcCSF (dark blue) and iCSF scans (light blue) were planned at the same fourth ventricle location with the same slice thickness (3 mm). **A**) Group average time series (*n* = 3) in the fourth ventricle ROI. The paced breathing task (5–5) starts after the first 30 s. Shaded area indicates the standard deviation. **B**) CSF signal spectra, filtered to remove the cardiac frequency, showing a clear peak at 0.1 Hz, the respiratory task frequency. Unfiltered CSF spectra are shown in Figure S2

Figure 3A and B shows the mean (± SD) timeseries and power spectra of the interleaved pcCSF and iCSF-data, both measured at the same location in interleaved repetition order during paced breathing. Both signal traces similarly show a CSF inflow effect lagging concurrent with the paced breathing trace from the respiratory belt. It is visible that signal fluctuations of both the pcCSF and iCSF increase with the start of the paced breathing task (at *t* = 30 s). The power spectra show a clear peak at the paced breathing task frequency (0.1 Hz) for both scan types.

**Fig. 4:**
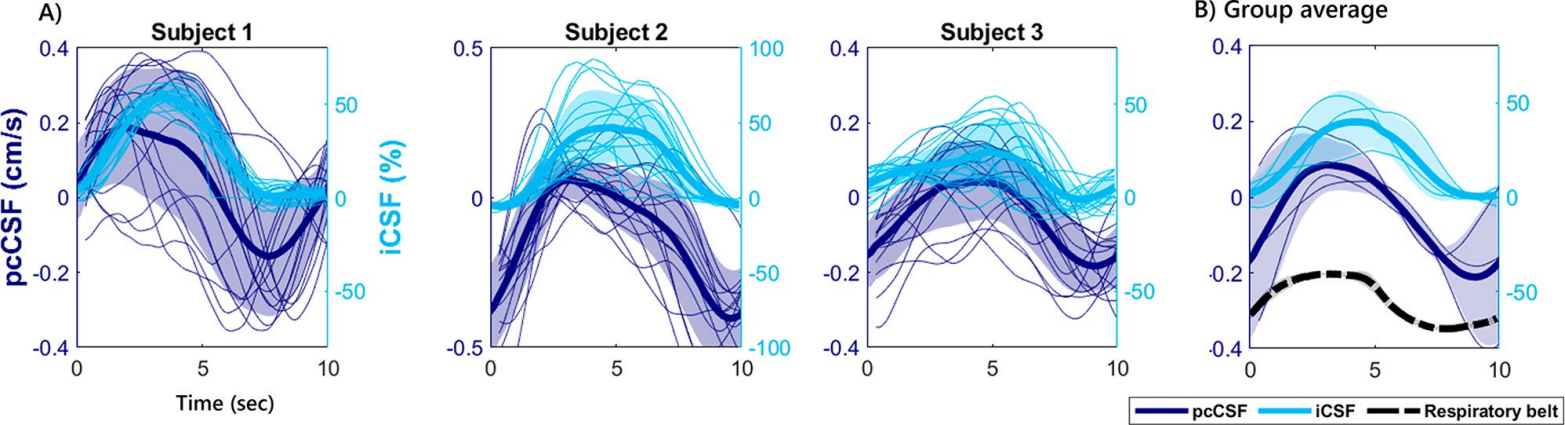
Individual results of fourth ventricle CSF dynamics during paced breathing. **A**) pcCSF (dark blue) and iCSF (light blue) for every single breathing cycle (thin lines) and participant average (thick lines) for the three participants (number of cycles = 15). **B**). The group average of the pcCSF, iCSF. Shared area indicates standard deviation across breathing cycles in **A**) and across participants in **B**). Net average flow over the breathing cycle can be seen, possibly explained by a background phase offset (see Discussion)

The averaged response over the ten-second breathing cycle window of all participants is visualized in Fig. 4A and B. This graph better illustrates that the iCSF method is only sensitive to cranial CSF inflow and then returns to a baseline during the outflow period. Instead, pcCSF captures bidirectional flow and can therefore also measure CSF outflow. Moreover, the pcCSF trace in Fig. 4B has more a pronounced in- and outflow curve and shows an earlier rise in CSF-inflow compared to iCSF. The breathing pattern was very similar between participants as seen by the low standard deviation on the respiratory belt traces.

### Experiment 2; BOLD-CSF coupling comparison between pcCSF and iCSF

**Fig. 5:**
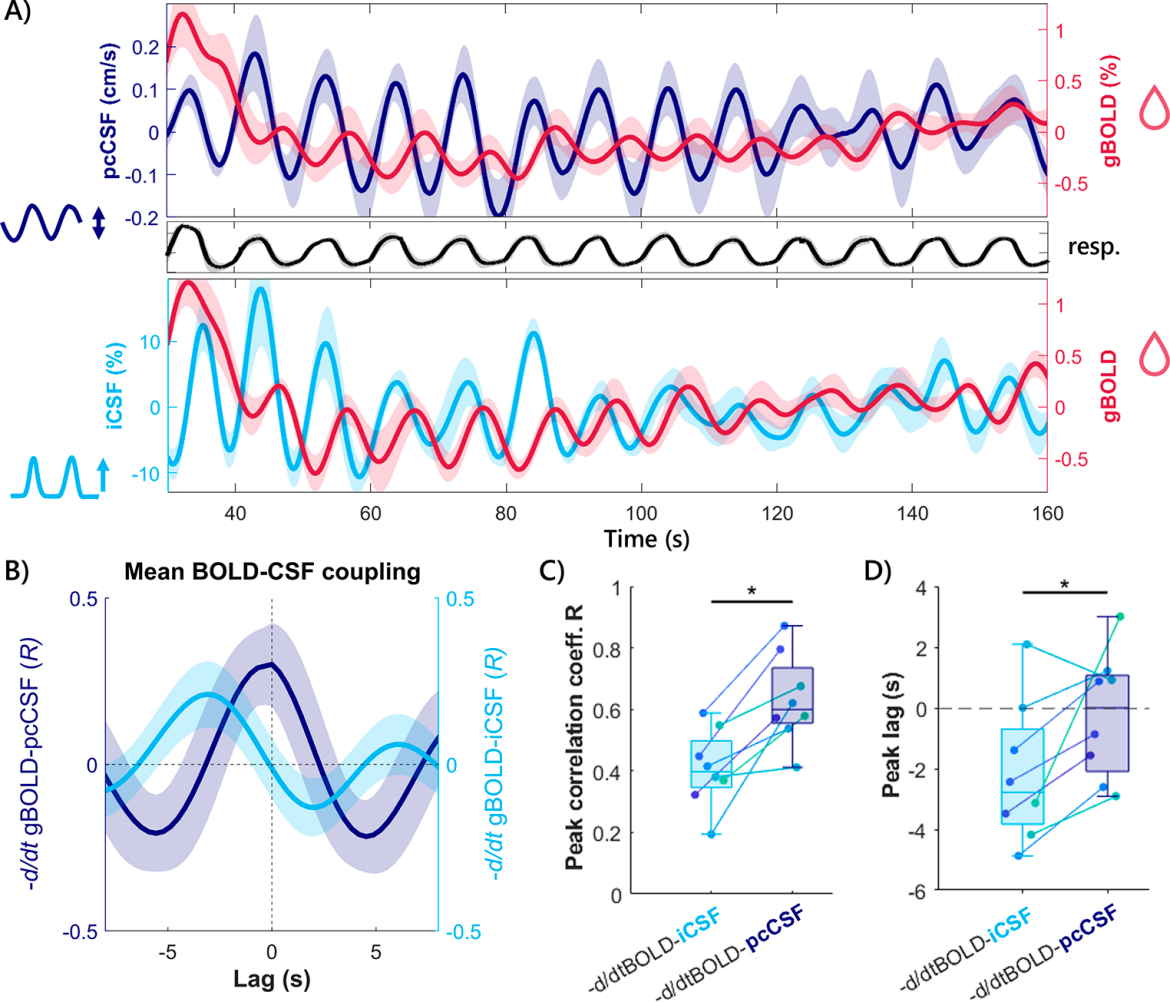
Results of $\:-\frac{d}{dt}gBOLD$-CSF coupling. **A**) Group average time series for pcCSF, iCSF and the corresponding gBOLD signal during paced breathing (start at 30 s), black line is the mean trace of the paced breathing paradigm. pcCSF might be affected slightly by respiration induced background phase offsets (see Discussion). **B**) Group average $\:-\frac{d}{dt}gBOLD$-CSF coupling (*n* = 8). Shaded area indicates standard error of the mean over participants. **C**) Participant specific peak correlation coefficient (R). **D**) peak lag, compared between $\:-\frac{d}{dt}gBOLD$-iCSF coupling and $\:-\frac{d}{dt}gBOLD$-pcCSF coupling. Significantly higher correlation coefficient and shorter lag is found for $\:-\frac{d}{dt}gBOLD$-pcCSF

Having assessed that pcCSF provides bidirectional CSF dynamics with a more pronounced in- and outflow effect than the original iCSF method, our next aim was to apply pcCSF measurements interleaved with gBOLD to assess BOLD-CSF coupling. Figure 5A displays the average gBOLD and CSF signal traces for both methods. As shown, both pcCSF and iCSF oscillate in a time-lagged pattern after each gBOLD peak, as subsequently summarized in a mean cross-correlation plot (Fig. 5B). This plot visualizes $\:-\frac{d}{dt}gBOLD$ and CSF signal coherence, averaged across participants, in both correlation magnitude (*R*) and lag (in seconds) between the sliding CSF signal and the fixed $\:-\frac{d}{dt}gBOLD$ signal.

To test for differences in peak *R* magnitude and lag, each participant’s peak *R* per scan was isolated. The peak *R* across participants was 0.41 ± 0.12 for $\:-\frac{d}{dt}gBOLD$-iCSF and 0.63 ± 0.15 for $\:-\frac{d}{dt}gBOLD$-pcCSF (Fig. 5C). The peak *R* was significantly higher for $\:-\frac{d}{dt}gBOLD$-pcCSF compared to $\:-\frac{d}{dt}gBOLD$-iCSF (*mean diff. =* 0.22, *signed rank* = 0, *p =* .0078), meaning that pcCSF produces higher peak signal coherence to $\:-\frac{d}{dt}gBOLD$ than iCSF. The interindividual variability of peak magnitude is similar between scans, as shown by similar standard deviations (0.12 vs. 0.15).

Besides the magnitude of the peak *R* per scan, the lag of this peak per participant was significantly closer to zero for $\:-\frac{d}{dt}gBOLD$-pcCSF coupling than for $\:-\frac{d}{dt}gBOLD$-iCSF coupling (*mean diff. =* 1.9 s, *signed rank =* 1, *p =* .016): the mean $\:-\frac{d}{dt}gBOLD$-iCSF cross-correlation coefficient lag was −2.17 ± 2.31 s, whereas for $\:-\frac{d}{dt}gBOLD$-pcCSF the mean lag was −0.24 ± 2.07 s.

## Discussion

In this study a new approach was introduced to study the coupling between CSF flow measured in the fourth ventricle and global cortical BOLD fluctuations. In contrast to the original approach, the main advantages of phase contrast MRI to track CSF flow are that it provides a quantitative measure of CSF flow and that it is sensitive to CSF outflow. To illustrate the properties of this new approach we performed two different experiments.

**Fig. 6:**
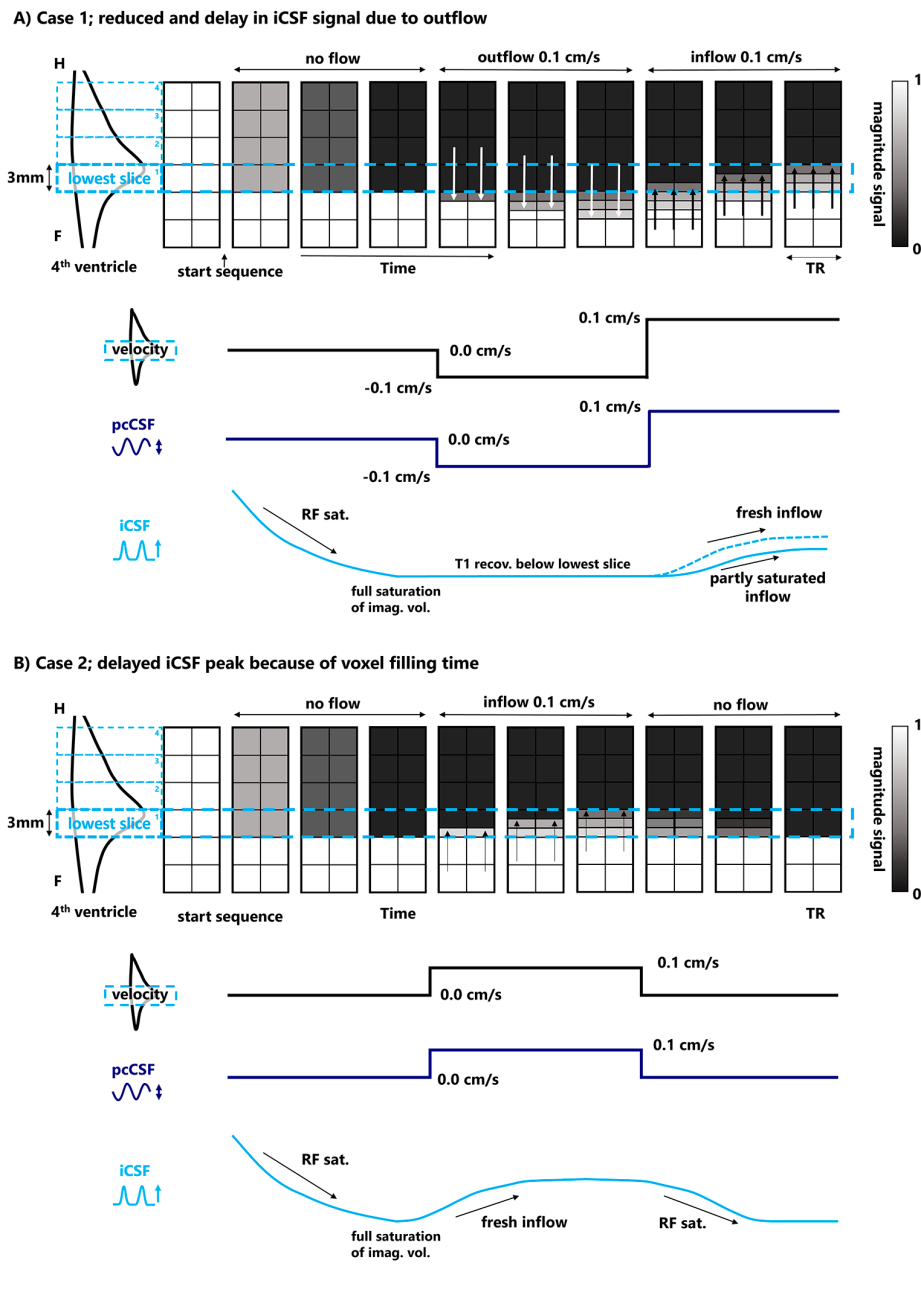
Schematic to explain iCSF signal delays in the bottom EPI slice compared to pcCSF. **A**) Case 1; CSF flowing from below back into the bottom slice that has experienced prior excitation pulses will delay and smear out the iCSF signal amplitude compared to fully relaxed inflowing spins. **B**) Case 2; the speed of CSF is rather slow compared to the TR (approximately 0.5 s, here shown per 1.0 s) and slice thickness (3 mm) of the BOLD scans. This implies that is takes several repetitions to fully refill the voxels in the bottom slice with less saturated spins. Moreover, the same delay of full replacement holds for outflow, both contributing to a smeared out iCSF response and complicating the interpretation of the iCSF signal changes. Colors in the blocks indicate the longitudinal magnetization that is present in the voxel at a given time, illustrating the voxel intensity differences seen on iCSF scans (though the CSF signal is always hyperintense to a degree). In contrast to iCSF, pcCSF in this schematic shows “instantaneous” signal changes related to its direct flow measurements

In the first experiment (Fig. 1), the phase contrast measurement (pcCSF) was performed at the same location as the original approach, which tracks the inflow of CSF by means of fast BOLD scanning (iCSF). Our results demonstrate that pcCSF shows an earlier rise in CSF-inflow compared to the iCSF signal (Fig. 4B). This can be understood by the fact that iCSF is based upon an inflow effect of unsaturated CSF spins, which will take some time to provide a maximum intensity change for two reasons. First, CSF spins flowing from below, back into the bottom slice, might have been excited by previous radiofrequency pulses from prior CSF outflow (Fig. 6A). This implies that when magnetization of the inflowing CSF is not yet fully relaxed, it could delay the maximum iCSF signal peak. Secondly, the speed of CSF is rather slow (on the order of 0.05 cm/s) compared to the TR (approximately 0.45 s) and slice thickness (3 mm) of the BOLD scans. It will therefore take several repetitions, and thereby several excitation pulses, to fully replace the voxel with lesser- or unsaturated inflowing CSF spins (Fig. 6B, during inflow). After the inflow of unsaturated spins stops, it will also take several excitations pulses to fully saturate the imaging slice, again prolonging the measured response (Fig. 6B, after inflow). Both effects could lead to a lag in the iCSF signal compared to the pcCSF measurement and smear out its CSF inflow trace over time. Additionally, this implies that the iCSF signal response depends on slice thickness (with thinner slices reaching peak signal change faster), which might complicate comparison across studies. Therefore, pcCSF could be considered a better reflection of BOLD-CSF coupling than methods based on a short-TR BOLD scan.

In the second experiment (Fig. 2), the BOLD-CSF coupling was compared between the standard iCSF scan and the novel pcCSF interleaved with cortical BOLD. We showed that the peak correlation coefficient was higher for BOLD-pcCSF compared to the BOLD-iCSF coupling (Fig. 5C). The higher correlation was expected as the sensitivity of pcCSF to bidirectional flow produces an oscillating signal that better fits to the full BOLD derivative, in contrast to iCSF where the negative BOLD derivatives were set to zero because of unidirectional flow. iCSF exhibited a more negative peak lag compared to pcCSF, which might again be explained by the two effects mentioned in the previous paragraph and illustrated in Fig. 6.

As shown in Fig. 2, the brain coverage for the cortical BOLD scan which is interleaved with the pcCSF measurement was smaller (9 slices) than the BOLD-iCSF scan (21 slices). This reduction in slice count was needed to shorten the TR (144 ms) to achieve a high enough temporal resolution when interleaved with the 204–300 ms pcCSF measurement. This smaller coverage of the BOLD scan is the main drawback of the interleaved approach. Differences in coverage might induce small changes in the averaged gBOLD signal between the two scan types, as different parts of the cortex are sampled. However, the smaller FOV was still adequate for extracting a robust gBOLD signal in our experiments as paced breathing gives a global rather than a local response in the cortex. Additionally, the slab can be freely moved to any region of interest to assure adequate coverage of the relevant parts of the cortex, when other stimuli are employed. As shown in Figure S1, in our experiments only minor differences in global BOLD signal changes could be observed when comparing the 21 slice and the 9 slice BOLD approach.

An overall slightly negative average net outflow can be observed in Fig. 4B. This might be of physiological origin since CSF can flow from the fourth ventricle towards the lower spine, where it can egress along the lumbar and sacral nerves [20, 21]. But there is a concern that slow flow measurements can be affected by a potential static background phase offset between the subtracted bipolar gradient pairs in the phase contrast measurements, which could be a potential limitation of this study [22]. However, the use of a fast field echo read-out in this study, instead of a single shot EPI read-out, minimized the delay time between two different velocity encodings used for background field removal. We therefore argue that the fluctuations measured using pcCSF might be slightly affected by respiration induced background phase offsets but are not driven by it. Phantom validation and repeated scanning could help to robustly measure such a net outflow effect in the future.

A low-pass frequency filter was applied to isolate slow paced-breathing induced CSF flow and low-frequency BOLD responses (<0.1 Hz) similarly as in [2, 6]. In order to translate this method to higher frequency effects (e.g. cardiac-driven CSF flow), the sampling frequency of the current approach should be increased as it is at the moment too close to the cardiac Nyquist frequency. Improvements in reconstruction of the real-time phase contrast acquisition (e.g. compressed SENSE [9], low-rank or AI approaches) could enable more undersampling to further speed up acquisition.

As listed in the methods, Experiment 1 and 2 have slightly different acquisition parameters, notably a longer TR mainly resulting from a lower V_enc_. This change was made to adjust for variable CSF velocities between participants: a lower V_enc_ produces scans with a higher velocity-to-noise ratio, though at the cost of increased gradient strength, longer TR, and increased risk of phase wrapping at peak in- or outflow. A part of Experiment 2 was carried out prior to Experiment 1, hence the change to lower V_enc_ mid-way in the study.

Our study makes use of a specific patch of the Philips software that allows for scan interleaving [13], which might not be supported by other software releases or vendors. Open pulse-programming environment such as Pulseq [23] and gammaSTAR [24] could provide more easy environments to program such interleaving approaches in while aiding study replicability across centers.

## Conclusion

By interleaving real-time phase contrast (pc)CSF flow measurements with cortical BOLD, with a combined dynamic scan time under 450 ms, pcCSF was shown to accurately capture CSF inflow and outflow whereas iCSF only captures the inflow component. Furthermore, BOLD-pcCSF coupling resulted in a higher signal coherence between BOLD and CSF signals, as well as a shorter temporal lag, compared to BOLD-iCSF coupling. We therefore propose the interleaved BOLD-pcCSF method as a more quantitative alternative to BOLD-iCSF for the study of brain clearance drivers.

## Electronic Supplementary Material

Below is the link to the electronic supplementary material.

Supplementary Material 1

## Data Availability

The data that support the findings of this study are available from the corresponding author, LH, upon reasonable request.

## Abbreviations

BOLD: Blood oxygen level dependent
gBOLD: The global BOLD signal in the cortex
CSF: Cerebrospinal fluid
iCSF: CSF fluctuations as measured with the inflow method
pcCSF: CSF fluctuations as measured with phase contrast
MRI: Magnetic resonance imaging
PC: Phase contrast
RT: Real-time
ROI: Region of interest
TE: Echo time (ms)
TR: Repetition time (ms)
FOV: Field of view
EPI: Echo planar imaging
SMS: Simultaneous multi-slice
SPIR: Spectral presaturation with inversion recovery
V_enc_: Velocity encoding (cm/s)
$\:-\frac{d}{dt}gBOLD$: Negative derivative of the gBOLD signal
