# Supplementary material for: BOLD-CSF dynamics assessed using real-time phase contrast CSF flow interleaved with cortical BOLD MRI
Source: Fluids Barriers CNS. 2024 Dec 24;21:107. doi: 10.1186/s12987-024-00607-8 (PMC11669233; doi:10.1186/s12987-024-00607-8)

Supplementary:

Figure S1: Comparison of gBOLD of 9 slices BOLD scan (dark blue) to 21 slices BOLD scan (light blue).

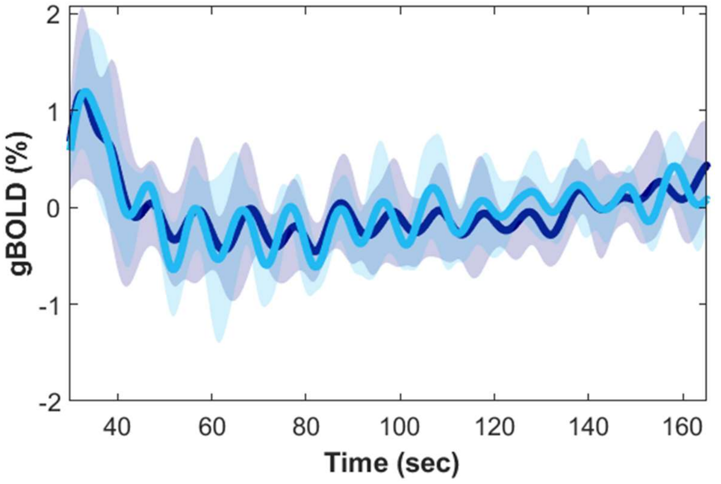

Figure S2: CSF power spectra. Unfiltered version of Figure 3B). showing the cardiac peak.

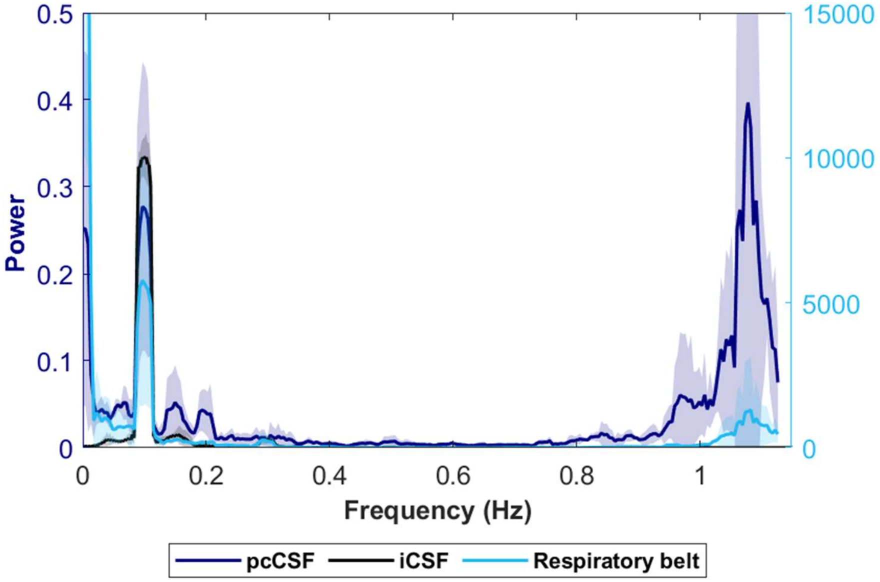

Supplement: Supplementary file 1 — Supplementary Material 1 [file 12987_2024_607_MOESM1_ESM.pdf]
